# Is there are relationship between polymorphisms *TSHR* gene frequencies and genetic ancestry markers in patients with Primary Congenital Hypothyroidism?

**DOI:** 10.1590/1678-4685-GMB-2024-0147

**Published:** 2025-10-27

**Authors:** Erik Artur Cortinhas-Alves, Victor Henrique Botelho Lourenço, Andreza Juliana Moreira da Costa, Ney Pereira Carneiro dos Santos, Leiliane Cruz Reis, Luiz Carlos Santana da Silva

**Affiliations:** 1 Universidade do Estado do Pará, Departamento de Morfologia e Ciências Fisiológicas, Belém, PA, Brazil.; 2 Universidade Federal do Pará, Programa de Pós-Gradução em Farmacologia e Bioquímica, Belém, PA, Brazil.; 3 Universidade Federal do Pará, Programa de Pós-Graduação em Oncologia e Ciências Médicas, Belém, PA, Brazil.; 4 Universidade Federal do Pará, Instituto de Ciências Biológicas, Laboratório de Erros Inatos do Metabolismo. Belém, PA, Brazil.

**Keywords:** Ancestry-informative markers, primary congenital hypothyroidism, TSHR gene, polymorphisms, genetic ancestry

## Abstract

The literature shows a correlation between ethnicity and pathogenic variants of the thyroid stimulating hormone receptor (*TSHR*) gene. Some of these polymorphisms may be risk factors for the development of primary congenital hypothyroidism (PCH). In this study, we investigated the relationship between the frequency of *TSHR* gene polymorphisms and the genetic influence of African, Amerindian, and European ancestry-informative markers in patients from an Amazonian population in Brazil who were diagnosed with PCH. The study was conducted on samples from 106 patients who were diagnosed with PCH. Genomic DNA was isolated from peripheral blood samples, and 10 exons from the *TSHR* gene were automatically sequenced. Ancestry-informative marker identification was performed using a panel of 48 markers, and the results were compared with parental Amerindian, Western European, and Sub-Saharan African populations using Structure v2.3.4 software. Four nucleotide alterations were identified among 49 patients. The distribution of tested ancestry markers among the 106 patients indicated a significant difference in the percentages of Amerindian (25.90 %), European (41.80 %), and African (32.20 %) ancestry. Logistic regression analysis revealed no significant association between the rs2075179 and rs1991517 polymorphisms and genetic ancestry. This study revealed no evidence of a relationship between polymorphic *TSHR* gene variants and genetic ancestry markers in patients with PCH.

## Introduction

The incidence of certain polymorphic variants of the thyroid-stimulating hormone receptor (*TSHR*) (UniProt: P16473) gene associated with the occurrence of PCH has been reported in different populations and ethnic groups (Narumi et al., 2009; Alves et al., 2010; Kollati et al., 2020; Da et al., 2021; Begum et al., 2023). Additionally, studies have identified a relationship between ethnicity and pathogenic TSHR gene variants (Chang et al., 2012; Da *et al.,* 2021; Narumi *et al.,* 2009).

Congenital hypothyroidism is one of the principal diseases that is detected in newborn screening worldwide, and most diagnoses are primary congenital hypothyroidism (PCH) (OMIM: 275200) (Wassner, 2018; Lau et al., 2020). Several epidemiological studies have been conducted worldwide (Stoppa-Vaucher et al., 2011; Garmendia Madariaga et al., 2014; Olmos et al., 2015; Taylor *et al.,* 2018), and the findings have demonstrated a relationship between ethnic variations and susceptibility to the development of PCH (Stoppa-Vaucher *et al.,* 2011). However, a limitation of these studies is that they did not consider ethnic or racial contributions based on genetic ancestry studies (e.g., INDEL polymorphisms).

In this context, INDEL (insertion/deletion) polymorphisms used as ancestry-informative markers (AIMs) are valuable tools for describing or confirming the historical formation of a population (Manta et al., 2013). As an alternative to self-reported racial and ethnic declarations, these markers are more precise in evaluating these contributions, especially in mixed populations such as the population of Brazil (Santos et al., 2010; Ramos et al., 2016).

Considering the complexity of genetic composition in mixed populations and the role of genetic polymorphisms in disease predisposition (Da Silva et al., 2017; Leal et al., 2020), we hypothesized that variations in different ethnic contributions might influence the frequency of *TSHR* gene polymorphisms. In this study, we evaluated the genetic contribution of African, Amerindian, and European AIMs (INDEL polymorphisms) and correlated these contributions with the frequencies of different TSHR gene polymorphisms in patients from a Brazilian Amazon population who were diagnosed with PCH.

## Methods

### Study population

This study was conducted with 106 patients (28 male patients and 78 female patients) who were diagnosed with PCH and treated at the Maternal-Infant and Adolescent Specialized Reference Unit (UREMIA/PA) in the city of Belém, in the state of Pará, northern Brazil.

### Inclusion criteria

The criteria for including patients in the study was a confirmed diagnosis of PCH based on clinical symptoms present at the first consultation and biochemical data (plasma TSH and T4 levels). TSH reference values with an upper cutoff of 20 mIU/L were used (Van Trotsenburg et al., 2021).

### Amplification and genotyping of the TSHR gene

Genomic DNA was isolated from peripheral blood samples of the patients using the Invisorb® Spin Blood Mini Kit (Invitrogen of Brazil Ltda., São Paulo, SP, Brazil). The *TSHR* gene coding region was amplified by PCR using a MJ96+/MJ96G thermocycler (Applied Biosystems of Brazil Ltda., São Paulo, SP, Brazil) and sequenced with the primers listed in Table 1 (De Roux et al., 1996).

**Table 1 -:** Primers for Amplification of TSHR Gene Exons.

Exon	Primers	Amplicon size (bp)	Amplicon Tm (ºC)
1	1F GAGGATGGAGAAATAGCCCCGAG	302	54
	1R CACTACTTCGGGCTGTTATTGAG		
2	2F TAAGGTGAATTATTAGAAAAGC	205	48
	2R CTTGATAGAACACGTTTAGAGAA		
3	3F GCAGAATCCATGAGGGTTGT	304	54
	3R AGAAACCAGGCCTCCCATTG		
4	4F ACCCTGTGGCGTAAATGCATAT	329	52
	4RCCCGACCCAGGCTATACACCATT		
5	5F GCTTTACTTATCTTCAACCTACC	291	52
	5R AGTTTGACTACAGGTTGTCTTC		
6	6F TATTGTGTCCTGTTATTTAAGTGCATA	293	54
	6R GTACTCTATAGAGTATATATGATAAGG		
7	7F TGGGATACATATGTGGGACCTG	324	54
	7R TGTTGGGTCACACTAACTCTGG		
8	8F TGGTCACATTTTATTCTGATATTTGT	272	54
	8R CTCCCCTTAATGTCTCCATTTATTCC		
9	9F TCATCTCCCAATTAACCTCAGG	408	54
	9R GCTTCCAATTTCCTCTCCAC		
10	FA ACTGTCTTTGCAAGCGAGTT	875	54
	RB GTGTCATGGGATTGGAATGC		
	FC TGGCACTGACTCTTTTCTGT	868	56
	RD GTCCATGGGCAGGCAGATAC		

PCR amplification was performed with 20 ng of genomic DNA in a final volume of 10 μL. The reaction included 1 × PCR buffer (Invitrogen of Brazil Ltda., São Paulo, SP, Brazil), 0.2 mM of each dNTP, 1.5 mM MgCl_2_, 2 pmol of each primer, and 0.5 U Taq polymerase (Invitrogen^®^).

Thirty PCR cycles were performed after an initial denaturation step at 94 °C for 1 min. Each cycle consisted of 1 min at 94 °C (denaturation), 1 min for primer annealing (53 °C-61 °C) and an extension step for 1 min at 72 °C. A final extension was completed at 72 °C for 10 min. All the PCR-derived products were visualized on 1.8 % agarose gels.

Population genotypes were determined by sequencing using the standard BigDye Terminator v 3.1 Cycle Sequencing Kit. The products were analyzed on an ABI 3130 Applied Biosystems sequencer.

### Ancestry analyses - INDEL markers

The INDEL markers were selected for the ancestry study. The 48 ancestry markers that were used in this study have been previously validated (Santos et al., 2010; Francez et al., 2012). The INDEL markers are detailed in Table S1.

Following gene amplification, the samples were genotyped using an ABI PRISM 3130 Genetic Analyzer (Applied Biosystems), and the results were analyzed by the GeneMapper v3.2 software (Applied Biosystems). The ABIGS LIZ-500 ladder (Applied Biosystems) was used as a reference for INDEL identification. Standards of known size were included in each assay for quality control.

The admixture model assumes that each individual inherits part of his or her ancestral markers from ancestral populations. The results were plotted against the three parental populations within the Brazilian population for ancestry stratification, as described in Francez et al. (2012.)

### Statistical analyses

Frequencies of polymorphisms were calculated by dividing the number of alleles carrying the polymorphism by the total number of analyzed alleles. Allelic frequencies are presented as percentages, and continuous variables are presented as means ± standard deviations.

Structure v 2.3.4 software was used to estimate admixtures by applying a 50000 burn length and 100,000 MMC repetitions after burning, employing an allele frequency-independent model. The data were normalized and analyzed by MANOVA with SPSS 22 software (IBM SPSS 22.0 Statistics for Windows Armonk (NY): IBM Corp; 2013).

Statistical analyses were performed using the RStudio V4.0.3 (RStudio, Inc., Boston, MA, USA). Ancestry indices were compared between groups by the ANOVA. Multiple logistic regressions were applied to assess potential associations between polymorphism frequencies and genetic ancestry markers by estimating the odds ratios (ORs) and their 95 % confidence intervals (CIs). All reported p values are two-sided; p values < 0.05 were considered statistically significant.

### Ethics

The study was approved by the Ethics Committee of the Institute of Health Sciences of the Federal University of Pará - UFPA (CAAE: 66812122.1.0000.0018). The legal guardians of the patients were consulted before blood collection and interviews. Patients who agreed to participate signed a standard consent form. Participation in the study was voluntary, and participants were free to withdraw from the study without consequences.

Written informed consent was obtained from all participants prior to their inclusion in the study, in accordance with the ethical standards of the institutional and/or national research committee and with the 1964 Helsinki Declaration and its later amendments or comparable ethical standards.

## Results

Sequencing of the *TSHR* gene coding region revealed four nucleotide alterations: rs2234919 (c.154C > A; p.Pro52Thr), rs2075179 (c.561T > C; p.Asn187=), rs113951800 (c.1377G > A; p.Ala459=), and rs1991517 (c.2181C > G; p.Asp727Glu). A total of 49 patients presented one or more polymorphisms in the TSHR gene coding region. Among these, five patients had two nucleotide alterations: three patients had rs2075179 (p.Asn187=) and rs1991517 (p.Asp727Glu), one patient had rs113951800 (p.Ala459=) and rs1991517 (p.Asp727Glu), and one patient had rs2234919 (p.Pro52Thr) and rs2075179 (p.Asn187=). The determined allelic frequency data are detailed in Table 2.

**Table 2 -:** Polymorphism frequencies in patients with primary congenital hypothyroidism.

rsID	Protein change	Genotype frequency	Allelic frequency
rs2234919	p.Pro52Thr	4/106 (3.77 %)	1.89 %
rs2075179	p.Asn187=	28/106 (CT: 23.58 %) (CC: 2.83 %)	14.62 %
rs113951800	p.Ala459=	4/106 (3.77 %)	1.89 %
rs1991517	p.Asp727Glu	18/106 (17 %)	8.5 %

**Figure 1 -:**
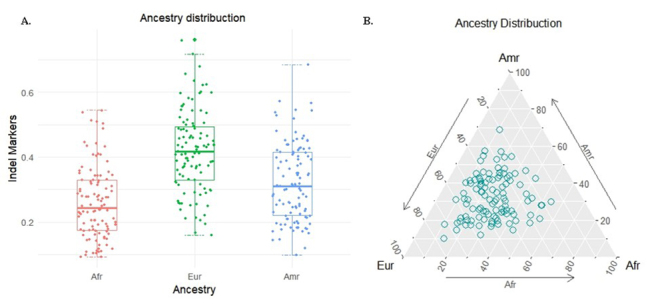
A. The boxplot compares the contributions of Amerindian (Amr), African (Afr), and European (Eur) markers in patients with primary congenital hypothyroidism (PCH). B. Estimates of genetic admixture of the patients.

The ancestry marker distribution of the 106 tested patients indicated a significant difference between Amerindian, European, and African ancestries (F=48.725; p<0.0001), as shown in Figure 1 A .

Figure 1 B  shows the ancestry profile for each patient. The closer one of the circles is to the vertices of the triangle, the greater the ancestry for that patient. This analysis allows for a visual representation of the different ancestries within the analyzed group of PCH patients.

Patients without polymorphisms were also tested (n=57). The ancestry markers in this group were significantly different between Amerindian, European, and African ancestries (F=22.599; p<0.0001). The ancestries of patients presenting any type of polymorphism were significantly different between European and African (F= 28.881; p<0.0001) and between European and Amerindian (F= 28.881; p<0.0001) ancestries, whereas no difference was observed between African and Amerindian (F= 28.881; p=0.341) ancestries. The European ancestry was more common in all the polymorphism subgroups (Table 3).

**Table 3 -:** Ancestry informative markers frequencies in patients with primary congenital hypothyroidism.

Patients	N	Genetic ancestry (Means±SD)			ANOVA test	Post-hoc
		African	European	Ameridian	F; p-value	Bonferroni
All patients	106	25.90±11.00	41.80±12.50	32.20±11.90	F=48.725; p<0.0001	African x European - p<0,001 African x Ameridian - p<0,001 European x Ameridian - p<0,001
Patients without polymorphisms	57	25.70±9.90	40.20±12.20	34.10±12.30	F=22.599; p<0.0001	African x European - p<0,001 African x Ameridian - p<0,001 European x Ameridian - p=0,016
All patients with any polymorphism	49	26.20±12.20	43.70±12.60	30.10±11.10	F=28.881; p>0.0001;	African x European - p<0,001 African x Ameridian - p=0,341 European x Ameridian - p<0,001
P52T	4	16.00±4.90	49.20±9.40	34.70±13.60	F=11.210; p=0.004	African x European - p=0,003 African x Ameridian - p=0,078 European x Ameridian - p=0,208
N187N	28	30.01±12.9	42.70±14.30	27.10±10.00	F= 12.274; p<0.0001	African x European - p<0,001 African x Ameridian - p=1 European x Ameridian - p<0,001
A459A	4	19.20±6.20	43.20±0.40	37.60±6.50	F=23.276; p<0.0001;	African x European - p<0,001 African x Ameridian - p=0,002 European x Ameridian - p=0,498
D727E	18	24.30±9.80	45.60±11.30	30.10±12.60	F=17,045; p<0.0001	African x European - p<0,001 African x Ameridian - p=0,387 European x Ameridian - p<0,001

**Figure 2 -:**
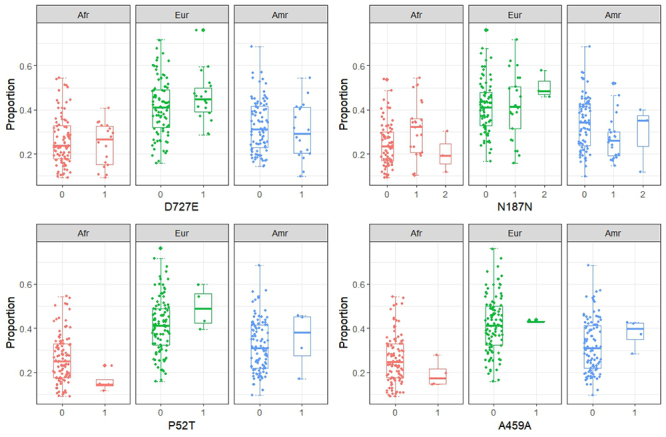
Distribution of the three ancestries and polymorphisms analyzed in this study, encompassing wild-type homozygous (0), heterozygous (1), and mutant homozygous (2). **A**. Polymorphism rs2234919 (P52T); **B**. Polymorphism rs2075179 (N187N); **C**. Polymorphism rs113951800 (A459A); **D**. Polymorphism rs1991517 (D527E).

No significant differences were observed in the logistic regression analysis regarding the relationship between the rs2075179 (p.Asn187=) (p=0.073) and rs1991517 (p.Asp727Glu) (p=0.2) polymorphisms and genetic ancestry (Figure 2). The rs2234919 (p.Pro52Thr) and rs113951800 (p.Ala459=) polymorphisms were not tested because of the small number of patients who exhibited these nucleotide changes.

## Discussion

To our knowledge, this is the first study to analyze the relationship between ancestry markers and frequencies of *TSHR* gene polymorphisms in patients with PCH from an Amazonian population. Additionally, this is the first study to characterize ancestry in patients with PCH in an Amazonian population.

Characterization of the genetic diversity and distribution of polymorphisms in different Amazonian populations might help identify specific risk markers for PCH. This is especially relevant in this region, where resources for diagnosis and treatment may be limited. Studies focused on admixed populations are scarce, and investigations of disease-associated polymorphisms in these populations represents a significant gap in scientific research.

All four nucleotide changes in the *TSHR* gene that were identified in this study were previously described in the region (Alves et al., 2010; Cortinhas-Alves et al., 2016). Our logistic regression analysis did not reveal significant associations between these polymorphisms and any type of ancestry. This could be explained by the heterogeneous genetic ancestry pattern of the analyzed sample or the limited sample obtained. Furthermore, Alves *et al.,* 2016 described the clinical findings of these polymorphisms; however, no associations could be established.

The frequencies of the identified polymorphisms were compared with those observed in other populations (Table S2) to establish potential relationships, and we obtained interesting findings. The rs2234919 polymorphism exhibited a low frequency in African and African American populations and a high frequency in some European populations; this pattern was also observed in our sample. Notably, our sample displayed a lower frequency than the sequencing data from the Brazilian population, which may be attributed to the underrepresentation of genomic data from individuals in the North/Pará region in these databases. Furthermore, the rs1991517 polymorphism presented the lowest frequency in our sample (Table S2), in contrast to other populations, where it was observed at a higher frequency.

Studies using self-reported race/ethnicity information have reported differences in the prevalence of PCH across various populations (Stoppa-Vaucher et al., 2011; Feuchtbaum et al., 2012). Compared with the European population, Asian and Latino (Hispanic) populations have higher rates of congenital hypothyroidism, whereas African populations have lower rates of congenital hypothyroidism (Hinton et al., 2010; Stoppa-Vaucher *et al.,* 2011; Feuchtbaum *et al.,* 2012).

Additionally, previous studies have corroborated an association between ethnic variations and genetic variants of the TSHR gene (Gabriel et al., 1999; Mühlberg et al., 2000; Sykiotis et al., 2003; Musa et al., 2008; Tug et al., 2012; Bayram et al., 2013). Evidence suggests a greater prevalence of PCH in populations with a lower degree of admixture and in Caucasian populations (Lohmueller et al., 2008). The literature indicates that genetic diversity in some African populations may protect these individuals from diseases such as thyroid dysgenesis (Schuster et al., 2010).

In a previous study that used self-reported race information, the prevalence of tireopaties in Brazil was lower among individuals with black skin than among individuals with white skin, and an intermediate prevalence was observed among individuals with brown skin (Sichieri et al., 2007). Olmos et al. (2015) investigated the influence of sex, race, and socioeconomic status on the diagnosis and treatment of thyroid disorders and concluded that individuals with brown and black skin were more strongly protected against hypothyroidism than were those with white skin.

In addition to the differences observed between Caucasian and African populations, differences in PCH rates among Asian populations have also been noted, reinforcing the importance of studies investigating the relationship between ethnicity and the presence of polymorphic *TSHR* gene variants (Park et al., 2016; Sun et al., 2018; Yu et al., 2018).

Moreover, literature on ancestry-informative markers in the Brazilian Amazonian population is scarce. Few studies have explored associations between AIMs and other diseases among patients with a specific ancestry in this region (Da Silva et al., 2017; Leal et al., 2020).

Our findings revealed a high prevalence of European ancestry in all the groups and subgroups that were analyzed and a low prevalence of African ancestry, especially in patients with the rs2234919 (p.Pro52Thr) polymorphism (Souza et al., 2019; Pena et al., 2020). However, this could be associated with the low number of carriers of this polymorphism in the sample (n=4).

In this context, our findings about ancestry markers in PCH patient profiles were similar to those reported in previous studies about ancestry contributions in Brazil, which revealed a lower prevalence of African ancestry than Amerindian and European ancestry (Manta et al., 2013). This ancestry profile was associated with the historical formation of the northern Brazilian population (Parra et al., 2003; Lins et al., 2010).

## Conclusion

The present study did not find evidence of a relationship between frequencies of allelic *TSHR* gene polymorphisms and genetic ancestry. However, several limitations should be noted, such as the diverse genetic origins of patients’ congenital hypothyroidism and the fact that not all genetic factors associated with PCH have been identified; thus, further studies on other genes associated with PCH is needed.

The INDEL analysis revealed differences between Amerindian, European, and African ancestry markers in the analyzed group, with a higher prevalence of Amerindian and European ancestry markers than African ancestry markers across all groups.

## Data Availability

The data are available at: https://figshare.com/s/81037ec2d7d1762dae0d, Published on April 15, 2025.

## Supplementary material

The following online material is available for this article:

Table S1 - Indel markers used in this study. Data adapted from Santos et al., 2010 and Francez *et al*. 2012.

Table S2 - Table adapted from the allele frequencies found in the study compared to other databases.
