# Supplementary material for: Is there are relationship between polymorphisms TSHR gene frequencies and genetic ancestry markers in patients with Primary Congenital Hypothyroidism?
Source: Genet Mol Biol. 2025 Oct 27;48(3):e20240147. doi: 10.1590/1678-4685-GMB-2024-0147 (PMC12560216; doi:10.1590/1678-4685-GMB-2024-0147)
Supplement: Table S1 - [file 1415-4757-GMB-48-03-e20240147-s1.pdf]

Genetics and Molecular Biology

https://doi.org/10.1590/1678-4685-GMB-2024-0147

Supplementary Material to "Is there are relationship between polymorphisms *TSHR* gene frequencies and genetic ancestry markers in patients with Primary Congenital Hypothyroidism?"

Table S1 - Indel markers used in this study. Data adapted from Santos et al., 2010 and Francez et al. 2012.

INDEL Identification		Short Alele Frequencies			Probability of Deviation From Hardy–Weinberg Equilibrium (p <0,05)
SNV's	Mark Identification	AFR	EUR	AME	
rs140762	Mid473	0.979	0.388	0.004	0.1415
rs1160871	Mid619	0.817	0.269	0.940	0.6016
rs2307644	Mid1448	0.074	0.465	0.008	0.1560
rs2308115	Mid1923	0.275	0.109	0.355	0.2405
rs1610941	Mid856	0.437	0.849	0.254	0.4353
rs16388	Mid99	0.786	0.439	0.990	0.0289
rs16383	Mid93	0.206	0.772	0.000	0.5919
rs1160910	Mid682	0.431	0.904	0.624	0.1658
rs2067128	Mid1039	0.175	0.513	0.154	0.7034
rs2307976	Mid1780	0.333	0.785	0.418	0.0819
rs2307666	Mid1470	0.135	0.593	0.008	0.0942
rs16416	Mid132	0.198	0.606	0.549	1.000
rs2067186	Mid1098	0.271	0.487	0.322	0.4631
rs2307754	Mid1558	0.074	0.607	0.428	0.4812
rs16654	Mid217	0.024	0.481	0.041	0.5735
rs140857	Mid568	0.119	0.439	0.076	0.3924
rs2307553	Mid1357	0.116	0.716	1.000	1.000
rs16710	Mid273	0.191	0.672	0.998	0.3284
rs2307880	Mid1684	0.476	0.462	0.145	0.1138
rs1610902	Mid818	0.758	0.266	0.015	0.4830
rs2067259	Mid1172	0.537	0.206	0.000	0.5386
rs2067263	Mid1176	0.913	0.309	0.242	0.1132
rs2307912	Mid1716	0.332	0.702	0.783	0.2328
rs2307554	Mid1358	0.383	0.853	0.905	0.0500
rs2307981	Mid1785	0.694	0.244	0.029	1.000
rs2067353	Mid1271	0.071	0.856	0.295	0.1389
rs1610866	Mid780	0.228	0.872	0.917	1.000
rs140783	Mid494	0.228	0.741	0.656	0.8519
rs140783	Mid625	0.320	0.675	0.845	0.0867
rs2307575	Mid1379	0.620	0.902	1.000	0.4878
rs2308203	Mid2011	0.197	0.734	0.817	0.1270
rs2307922	Mid1726	0.167	0.672	0.612	0.5897
rs2308144	Mid1952	0.163	0.291	0.845	0.5652

INDEL Identification		Short Alele Frequencies			Probability of Deviation From Hardy–Weinberg Equilibrium (p <0,05)
SNV's	Mark Identification	AFR	EUR	AME	
rs140765	Mid476	0.000	0.328	0.854	1.000
rs16712	Mid476	0.633	0.700	0.407	0.7088
rs16432	Mid152	0.249	0.191	0.942	0.1109
rs16635	Mid196	0.594	0.494	0.035	0.2654
rs1610864	Mid778	0.771	0.787	0.335	0.6748
rs2307799	Mid1603	0.088	0.391	0.979	0.2180
rs2307582	Mid1386	0.182	0.187	0.755	0.6971
rs1160894	Mid660	0.221	0.609	0.858	0.5584
rs140864	Mid575	0.108	0.000	0.652	1.000
rs16653	Mid216	0.739	0.853	0.368	0.2372
rs140770	Mid481	0.089	0.306	0.000	1.000
rs1610996	Mid913	0.006	0.003	0.101	0.1877
rs25574	Mid350	0.061	0.047	0.416	0.2150
rs1611070	Mid988	0.094	0.371	0.696	1.000
rs16460	Mid1184	0.279	0.368	0.733	0.2216
