# Supplementary material for: Is there are relationship between polymorphisms TSHR gene frequencies and genetic ancestry markers in patients with Primary Congenital Hypothyroidism?
Source: Genet Mol Biol. 2025 Oct 27;48(3):e20240147. doi: 10.1590/1678-4685-GMB-2024-0147 (PMC12560216; doi:10.1590/1678-4685-GMB-2024-0147)
Supplement: Table S2 - [file 1415-4757-GMB-48-03-e20240147-s2.pdf]

Genetics and Molecular Biology

https://doi.org/10.1590/1678-4685-GMB-2024-0147

Supplementary Material to "Is there are relationship between polymorphisms *TSHR* gene frequencies and genetic ancestry markers in patients with Primary Congenital Hypothyroidism?"

Table S2 - Table adapted from the allele frequencies found in the study compared to other databases.

rsIDs	Allele frequency of this study*	Allele frequency African/African American**	Allele frequency Admixed American**	Allele frequency Ashkenazi Jewish**	Allele frequency East Asian**	Allele frequency European (Finnish)**	Allele frequency Middle Eastern**	Allele frequency European (non-Finnish)**	Allele frequency Amish**	Allele frequency South Asian**	Allele frequency Remaining**	Brazilian frequency***
rs2234919	0.0189	0.01284	0.02988	0.1257	0.008802	0.05095	0.1117	0.05724	0.2061	0.1987	0.06867	0.046114
rs113951800	0.1462	0.004586	0.008297	0.01874	0.0009807	0.01306	0.03415	0.02300	0.04725	0.04056	0.02028	0.016652
rs2075179	0.0189	0.5475	0.1334	0.1445	0.3238	0.1739	0.1683	0.1313	0.2314	0.1628	0.1689	0.209223
rs1991517	0.085	0.9384	0.8672	0.9053	0.8472	0.8473	0.9335	0.9173	0.8651	0.9126	0.9098	0.917165

*Data from this study, **Data from Chen et al., 2024, *** Data from Naslavsky et al., 2022.

References

Chen S, Francioli LC, Goodrich JK et al. (2024) A genomic mutational constraint map using variation in 76,156 human genomes. Nature 625:92-100.

Naslavsky MS, Scliar MO, Yamamoto GL, Wang JYT, Zverinova S, Karp T, Nunes K, Ceroni JRM, Carvalho DL, Simões CES *et al.* (2022) Whole-genome sequencing of 1,171 elderly admixed individuals from Brazil. Nat Commun 13:1004.
